# Troponin elevation in patients with various tachycardias and normal epicardial coronaries

**Published:** 2008-08-01

**Authors:** Khalil Kanjwal, Naser Imran, Blair Grubb, Yousuf Kanjwal

**Affiliations:** University of Toledo Medical Center. Toledo OH

**Keywords:** acute coronary syndrome, supraventricular tachycardia

## Abstract

Troponin elevation is usually synonymous with acute coronary syndrome (ACS). Although sensitive for ACS, the elevation of serum troponin, in the absence of clinical evidence of ischemia, should prompt a search for other etiologies of myocardial necrosis. In fact, elevated values of troponin are correlated with myocardial necrosis even though it does not discriminate the mechanism involved. We report a series of seven patients (age range 18-67 years), who presented with complaints of chest discomfort and were found to have regular supraventricular tachycardia (5 patients) and one patient each with atrial fibrillation and ventricular tachycardia. All these patients had elevated troponin I and underwent coronary angiography that revealed normal epicardial coronary arteries. This is first case series in which all patients underwent coronary angiography and none of the patients was hemodynamically unstable at the time of presentation. Patients with elevated troponin due to conditions other  than ACS can receive inappropriate and delayed definitive diagnosis and treatment.

## Introduction

Troponin elevation usually signifies some myocardial damage and is associated with significant obstructive coronary artery disease. There are multiple conditions that can cause elevation of troponin besides myocardial ischemia like sepsis, renal failure, decompensated heart failure, pulmonary embolism, coronary vasospasm, prolonged hypotension and tachycardias associated with hypotension to mention few. Although there have been multiple case reports of troponin elevation associated with tachycardias,  coronary artery disease was ruled out either by stress test or by coronary angiography. We report a case series of 7 patients with different kinds of tachycardias and troponin elevation who underwent coronary angiography and were hemodynamically stable.

## Method

Six patients of supraventricular tachycardias of different duration and one case of sustained ventricular tachycardia were found in our database. All cases presented with palpitations and chest discomfort of different duration. Patients underwent routine laboratory investigations and all of them had two-dimensional echocardiography. All patients were found to have elevated troponin I. There were three cases of accessory pathway mediated tachycardia (AVRT), two cases of atrioventricular-nodal reentrant tachycardia (AVNRT) and one case each of atrial fibrillation (AF) with fast ventricular response and ventricular tachycardia (VT). All patients underwent coronary angiography and had normal coronary arteries. Five patients (3 AVRT and 2 AVNRT) underwent successful radiofrequency ablation. One with atrial fibrillation was subsequently cardioverted after coronary angiography and placed on sotalol. The patient who presented with ventricular tachycardia was placed on amiodarone and had a biventricular ICD implanted for non-ischemic cardiomyopathy. All patients had elevation of Troponin I (Normal 0.00-0.04 ng/ml). Table 1

## Discussion

The joint committee of the European Society of Cardiology, the American College of Cardiology, and the American Heart Association has accepted the measurement of troponin T and I in serum as the standard biomarker for the diagnosis of acute myocardial infarction and for diagnosis and management of acute coronary syndromes [1-3]. However, the ACC/AHA guidelines also indicate that the myocardial necrosis signified by troponin elevation may not necessarily be due to atherosclerotic coronary artery disease and that myocardial infarction should therefore be diagnosed in conjunction with other supportive evidence. There have been few reports of troponin elevation in various tachyarrythmias. Bakshi [4] and colleagues in his study of 21 patients with troponin elevation with normal angiogram found that tachycardia was the culprit in 28% of patients. Zellweger [5] and associates reported 4 patients with supraventricular tachycardia who were found to have elevated troponin levels without evidence of coronary artery disease. However, in their report, only two patients were taken for coronary angiography and two had stress tests. Redfearna etal [6], reports seven patients of troponin elevation with supraventricular tachycardia. All these patients underwent coronary angiography and had normal epicardial coronary arteries. However, in his report two patients had hemodynamic compromise. These reports illustrate that troponins can be released because of tachycardia alone in the absence of myodepressive factors, inflammatory mediators, and coronary artery disease. We believe our series of patients with troponin elevation from various tachycardias with angiographically proven normal coronary vessels and without any hemodynamic compromise will be the largest one published till date.

All patients in our series underwent coronary angiography and had normal epicardial vessels. None of our patient was hemodynamically unstable and therefore the troponin rise in our series was a direct result of tachycardia.

The exact mechanism for troponin elevation during a tachycardia remains unknown. However, the most likely mechanism may be shortening of diastole with subsequent subendocardial ischemia [1]. Coronary perfusion especially to subendocardium occurs predominantly during diastole. Increase in heart rate causes diastole to shorten with subsequent decrease in subendocardial perfusion. Another, possible mechanism for tachycardia mediated troponin elevation is myocardial stretch. Higgins et al [7] and Qi et al [8] found a direct correlation between rise in cardiac BNP (B-Type Natriuretic Peptide) and troponins in patients with various tachycardias. This might be a limitation in some of the studies reported including ours, as BNP levels were not obtained. There was no relation of type and duration of the tachycardia with peak troponin elevation.

## Conclusion

The patients admitted within the framework of tachyarrhythmia without hemodynamic instability, the decision whether to obtain serum markers of myocardial ischemia should cover a comprehensive clinical context. Patients with elevated troponin due to conditions other than ACS can receive inappropriate and delayed definitive diagnosis and treatment.

## Figures and Tables

**Table 1:**
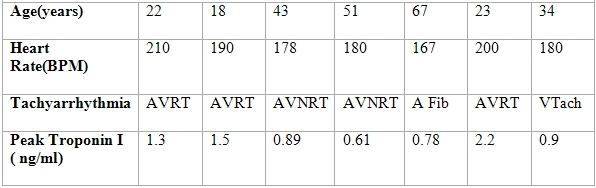
Baseline characteristics of the patients
